# Cardiogenic shock complicating multisystem inflammatory syndrome following COVID-19 infection: a case report

**DOI:** 10.1186/s12872-021-02304-y

**Published:** 2021-10-29

**Authors:** Michael I. Gurin, Yue J. Lin, Samuel Bernard, Randal I. Goldberg, Navneet Narula, Robert T. Faillace, Carlos L. Alviar, Sripal Bangalore, Norma M. Keller

**Affiliations:** 1 The Leon H. Charney Division of Cardiology, New York University Grossman School of Medicine, New York University Langone Medical Center, 462 First Avenue, NBV 17 South Room 5, New York, NY 10016 USA; 2 Department of Emergency Medicine, New York University Grossman School of Medicine, New York, NY USA; 3 Department of Pathology, New York University Grossman School of Medicine, New York, NY USA; 4 Department of Medicine, Albert Einstein College of Medicine/Jacobi Medical Center, Bronx, NY USA

**Keywords:** Acute heart failure, Cardiogenic shock, Multisystem inflammatory syndrome, SARS-CoV-2, Endomyocardial biopsy, Case report

## Abstract

**Background:**

With the high prevalence of COVID-19 infections worldwide, the multisystem inflammatory syndrome in adults (MIS-A) is becoming an increasingly recognized entity. This syndrome presents in patients several weeks after infection with COVID-19 and is associated with thrombosis, elevated inflammatory markers, hemodynamic compromise and cardiac dysfunction. Treatment is often with steroids and intravenous immunoglobulin (IVIg). The pathologic basis of myocardial injury in MIS-A, however, is not well characterized. In our case report, we obtained endomyocardial biopsy that revealed a pattern of myocardial injury similar to that found in COVID-19 cardiac specimens.

**Case presentation:**

A 26-year-old male presented with fevers, chills, headache, nausea, vomiting, and diarrhea 5 weeks after his COVID-19 infection. His SARS-CoV-2 PCR was negative and IgG was positive, consistent with prior infection. He was found to be in cardiogenic shock with biventricular failure, requiring inotropes and diuretics. Given concern for acute fulminant myocarditis, an endomyocardial biopsy (EMB) was performed, showing an inflammatory infiltrate consisting predominantly of interstitial macrophages with scant T lymphocytes. The histologic pattern was similar to that of cardiac specimens from COVID-19 patients, helping rule out myocarditis as the prevailing diagnosis. His case was complicated by persistent hypoxemia, and a computed tomography scan revealed pulmonary emboli. He received IVIg, steroids, and anticoagulation with rapid recovery of biventricular function.

**Conclusions:**

MIS-A should be considered as the diagnosis in patients presenting several weeks after COVID-19 infection with severe inflammation and multi-organ involvement. In our case, EMB facilitated identification of MIS-A and guided therapy. The patient’s biventricular function recovered with IVIg and steroids.

**Supplementary Information:**

The online version contains supplementary material available at 10.1186/s12872-021-02304-y.

## Learning points

- Identification and detection of MIS-A can expedite initiation of appropriate therapy.
- To understand the pathology and pattern of myocardial injury in the multisystem inflammatory syndrome following SARS-CoV2 infection.
- The mechanism of cardiac injury in MIS-A is likely a steroid-responsive process.

## Background

Multisystem inflammatory syndrome in adults (MIS-A) is a rare sequela of COVID-19 infection. Patients with MIS-A present with fevers, elevated inflammatory markers, evidence of current or prior COVID-19 infection, severe dysfunction of one or more extra-pulmonary systems, and absence of severe respiratory illness [1]. Myocardial injury is well described in COVID-19 infection [2, 3], however, less is known about the pathologic basis of myocardial injury in MIS-A. We present the case of a young male who presented several weeks after COVID-19 infection with cardiogenic shock from MIS-A. We obtained endomyocardial biopsy (EMB), helping identify the diagnosis and guide therapy.

## Case presentation

A 26-year-old male presented with 1 week of fevers, chills, headache, nausea, vomiting and diarrhea. Upon evaluation in the emergency department, he was febrile to 39.2 C, hypotensive to 75/35 mmHg, tachycardic at 126 beats per minute, with an oxygen saturation of 96% on 6 L nasal cannula. His labs were notable for leukocytosis (white blood cell count 17.1/nL), lymphopenia (absolute lymphocyte count 0.73/nL), acute kidney injury (creatinine 1.9 mg/dL), lactic acidosis (lactate 4.1 mmol/L), and elevated troponin levels (troponin T 0.218 µg/L, normal range < 0.09 µg/L). SARS-CoV-2 PCR was negative but SARS-CoV-2 IgG was positive. Point of care ultrasound showed severely reduced biventricular function. He was admitted to the cardiac care unit and given intravenous diuresis and inotropes for the management of cardiogenic shock (Additional file 1).

**Fig. 1:**
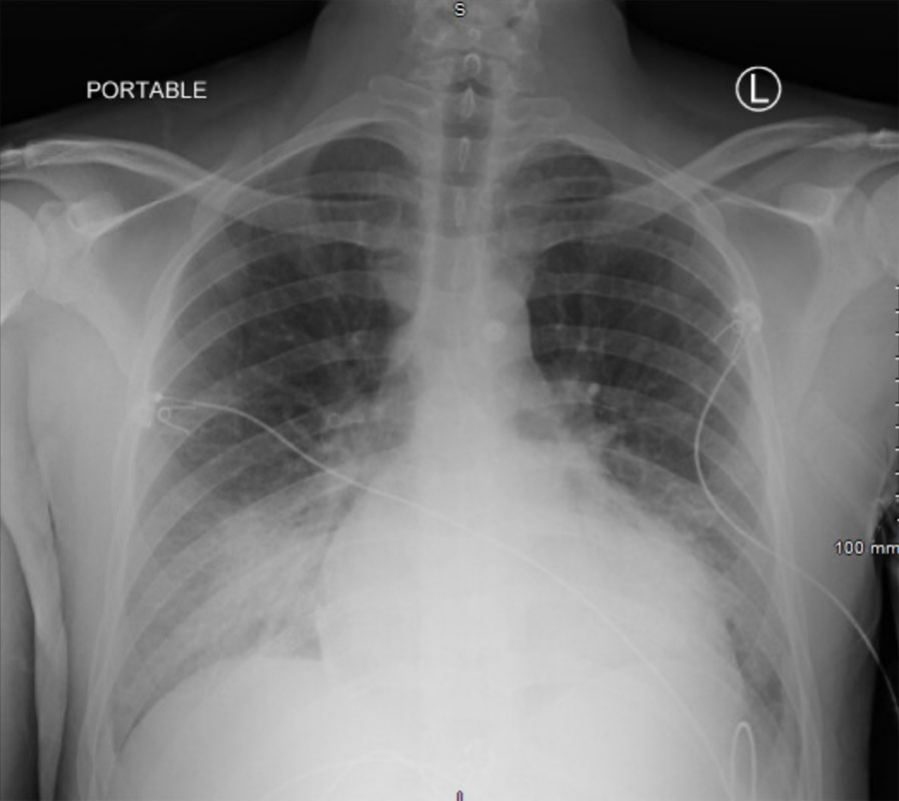
Chest radiograph. Hazy opacification of the right lower lung field and left lower lobe opacity. Cardiac silhouette is borderline in size

**Fig. 2:**
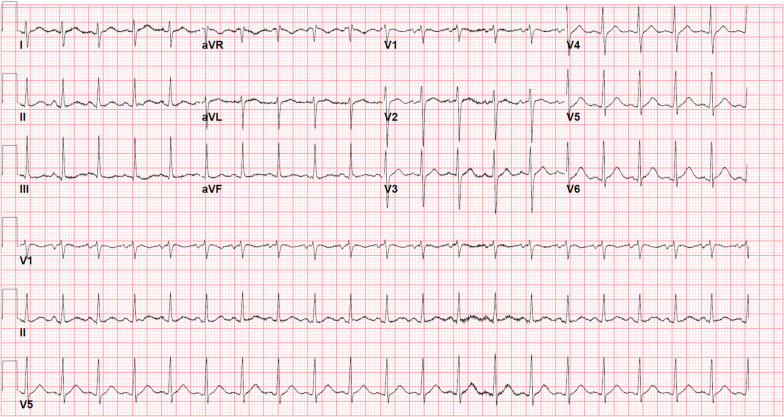
Electrocardiogram. Sinus tachycardia with right axis deviation

Further admission laboratory assessment was notable for elevations in transaminases, procalcitonin, NT-proBNP, ferritin, D-dimer, ESR, and C-reactive protein (Table 1). Chest x-ray showed opacification of the right lower lung field, a left lower lobe opacity, and borderline enlarged cardiac silhouette (Fig. 1). Electrocardiogram showed sinus tachycardia with right axis deviation (Fig. 2). An echocardiogram demonstrated a left ventricular ejection fraction of 20% with global hypokinesis, mildly dilated left ventricle, biatrial enlargement, hypokinetic and dilated right ventricle, moderate to severe mitral regurgitation due to leaflet tethering, severe tricuspid regurgitation, small pericardial effusion, and an estimated pulmonary artery systolic pressure of 53 mmHg with a right atrial pressure of 15 mmHg (Additional files 2 and 3 : Video 1).) .

**Table 1:** Admission laboratory test values

Admission laboratory tests	Results	Normal range
Complete blood count		
White blood cell count (× 10^3^/nL)	17.1	3.9–10.6
Hemoglobin (g/dL)	13.1	13.5–17.5
Hematocrit (%)	39.9	41–53
Platelets (× 10^3^/nL)	152	150–440
Basic metabolic panel		
Sodium (mEq/L)	136	135–145
Potassium (mEq/L)	3.4	3.5–5.0
Chloride (mEq/L)	98	98–108
Bicarbonate (mEq/L)	19	24–30
Blood urea nitrogen (mg/dL)	28	5–26
Creatinine (mg/dL)	1.9	0.5–1.5
Glucose (mg/dL)	176	70–111
Hepatic function panel		
Protein (g/dL)	6.0	6.0–8.5
Albumin (g/dL)	3.3	3.5–5.5
Bilirubin total (mg/dL)	1.2	0.1–1.2
Bilirubin direct (mg/dL)	0.5	0.0–0.2
Aspartate aminotransferase (U/L)	541	11–39
Alanine aminotransferase (U/L)	650	11–35
Alkaline phosphatase (U/L)	94	25–100
Coagulation profile		
Prothrombin time (s)	17.7	10.5–13.4
International normalization ratio	1.6	0.8–1.1
Activated partial thromboplastin time (s)	27.6	27.8–37.3
Venous blood gas		
pH	7.29	7.32–7.43
Pco_2_ (mm Hg)	46	41–54
Lactate (mmol/L)	4.1	0.6–1.4
Cardiac biomarkers		
N-terminal pro-B-type natriuretic peptide (pg/mL)	27,037	1–125
Troponin-T (µg/L)	0.218	≤ 0.09
Inflammatory markers		
Erythrocyte sedimentation rate (mm/hr)	42	0–10
C-reactive protein (mg/L)	250.5	0–3
D-dimer (ng/mL)	1149	0–230
Ferritin (ng/mL)	6084	22–322
Procalcitonin (ng/mL)	5.82	0.02–0.08

*Pco*_*2*_ partial pressure of carbon dioxide

On the evening of admission, bedside pulmonary artery catheter was placed with the following hemodynamics: RA 17 mmHg, RV 36/15 mmHg, PA 35/24 mmHg with mean 28 mmHg, PCWP 24 mmHg, mixed venous O_2_ saturation 53%, cardiac output 3.5 L/min, cardiac index 1.9 L/min/m^2^, system vascular resistance of 1440 dynes/s/cm^−5^. Cardiac output was calculated based on Fick’s formula using an assumed oxygen consumption. Pulmonary Artery Pulsatility Index (PAPi) was 0.7 (normal PAPi > 1.0 [4]), consistent with right ventricular dysfunction and biventricular shock.

Milrinone was initiated after PA catheter measurements were consistent with biventricular shock and intravenous diuretics were continued. In light of his ongoing hypotension, norephinephrine was initiated to maintain a MAP ≥ 65 mmHg. Given the patient’s ongoing cardiogenic shock and rapid decline, the decision was made to treat empirically for fulminant giant cell myocarditis and MIS-A as his COVID-19 diagnosis was 5 weeks prior. As such, he was empirically treated with 1000 mg of solumedrol and intravenous immunoglobulin (IVIg) therapy.

**Fig. 3:**
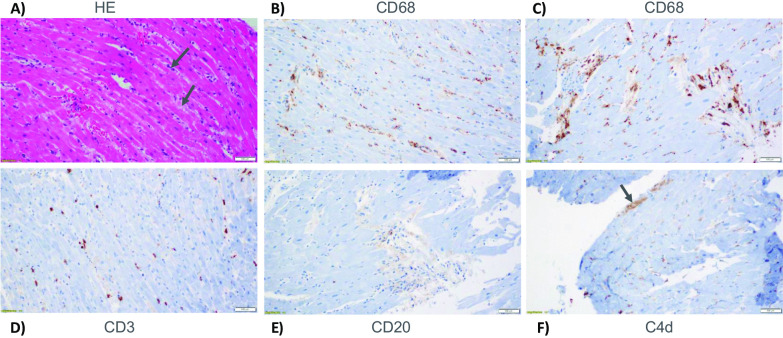
Histological assessment of endomyocardial biopsy. **A** Hematoxylin and eosin stain (HE) with arrows pointing to areas of edema seen as myocyte separation and bubbly nature of the interstitium. **B**, **C** Immunostain for macrophages (CD68) shows presence of significant interstitial macrophages. **D** Immunostain for T lymphocytes (CD3) show scattered lymphocytes with no evidence of myocyte damage. **E** Immunostain for B lymphocytes (CD20) shows rare positive cells. **F** Immunostain for C4d stain, a complement marker is positive focally in rare subendocardial myocytes (arrow) consistent with ischemic necrosis, probably secondary to right ventricular strain. (Magnification: **A**–**F**, 200×)

An endomyocardial biopsy was subsequently performed (Fig. 3). Histologic assessment showed interstitial edema and inflammatory infiltrate consisting predominantly of interstitial macrophages with scant T lymphocytes. Rare subendocardial myocytes with ischemic injury as highlighted by immunostain for C4d were seen. No eosinophils, giant cells or vascular thrombi were present. Given the macrophage predominant histology in combination with the remainder of his findings, a diagnosis of MIS-A was made. Steroid dosing was de-escalated to 2 mg/kg daily and IVIg was continued for 2 days.

Despite antibiotic therapy and improving hemodynamics, the patient remained hypoxemic on 6 L nasal cannula with a rising D-Dimer (8147 ng/mL). A computed tomography scan was performed on hospital day 6 demonstrating multiple bilateral segmental and subsegmental pulmonary thromboemboli. The patient was transitioned from prophylactic to therapeutic heparin with rapid resolution of hypoxemia. Bilateral lower extremity venous duplexes showed no evidence of deep venous thrombosis.

By hospital day 7, the patient was tapered off milrinone. He was transitioned to apixaban, and guideline-directed medical therapy was initiated with metoprolol succinate, sacubitril-valsartan and spironolactone. Repeat echocardiogram prior to discharge showed an LVEF 75% with normal RV size and function, no more than trace valvular regurgitation and a small pericardial effusion (Additional files 4 and 5: Video 2). His renal function returned to normal (0.9 mg/dL) without requiring renal replacement therapy and liver enzymes continued to downtrend.

The patient is doing well following discharge at 1 and 3 months with unlimited exercise tolerance and no evidence of arrhythmias or hemodynamic compromise. Repeat echocardiogram showed preserved biventricular function. He will have a cardiac MRI to further characterize the effect of myocardial injury and he is restricting vigorous exercise. He was referred to cardiac rehabilitation and remains on the same medications.

## Discussion and conclusions

Given the increased prevalence of SARS-CoV2 infections around the world, MIS-A is becoming a recognized entity. Our patient presented with new onset heart failure and cardiogenic shock several weeks after his COVID-19 infection, prompting the decision to pursue EMB. The results of the biopsy allowed the team to de-escalate steroid therapy and narrow our diagnosis to MIS-A. To date, this is the first case reported in the literature when the diagnosis of MIS-A was made on EMB as histology was not consistent with myocarditis [5–7].

Despite initial suspicion for acute fulminant myocarditis, the histology showed a macrophage infiltrate with few lymphocytes; a pattern of myocardial injury commonly identified in cardiac specimens from patients with COVID-19 [2, 3]. Although, inconsistent with myocarditis, the mechanism of heart failure in our patient remains unclear. In a case series of pediatric patients with multisystem inflammatory syndrome, it is suggested that heart failure in MIS-C is related to myocardial stunning and edema based on lower troponin elevations and the rapid resolution of systolic dysfunction [8]. Cardiac MRI of these patients show myocardial edema and hyperemia without late gadolinium enhancement suggestive of myocardial necrosis or fibrosis [9]. Similarly, the troponin elevation in this case was not markedly elevated with rapid resolution of left ventricular systolic function.

Additionally, the prothrombotic nature of MIS-A is demonstrated in this case given his multiple pulmonary thromboemboli. Given the right axis deviation on the patient’s ECG, persistent hypoxemia, and right ventricular myocyte necrosis on histology, these pulmonary emboli were almost likely present on his initial presentation. This increased risk of thrombosis is not entirely understood, but may be related to underlying coagulopathy, endothelial injury leading to decreased fibrinolysis and increased thrombin production, activation of the complement system, and the ensuing thrombo-inflammation similarly seen in COVID-19 infections [10]. Further, it remains unclear whether MIS-A/C is a distinct disease entity from COVID-19 or whether it exists on a spectrum of disease, given many overlapping features and similar patterns of myocardial injury.

Given the timing of his presentation several weeks after COVID-19 infection and his concomitant hemodynamic compromise, we pursued endomyocardial biopsy to evaluate for etiologies of acute myocarditis that tend to be steroid responsive (i.e. giant cell myocarditis) and that require establishing a diagnosis in a timely fashion. With improved recognition and detection of this clinical syndrome, endomyocardial biopsy may not be necessary to further guide management. Despite lack of consensus, limited evidence has demonstrated improvements in MIS-A with steroids and IVIg [8]. As such, these therapies were utilized in this case with recovery of biventricular function. This suggests the mechanism of injury is a sequela of inflammation. Additional data on this treatment strategy will provide further reassurance about proper triaging and management decisions.

The strength of this case report are its novel pathologic findings of MIS-A, which we believe will be of interest to the broader medical community. Further, the decision to start steroids early may have important prognostic value and guide decision-making in future cases of cardiac dysfunction associated with MIS-A. Even with vaccination rates increasing, new strains of SARS-CoV-2 continue to emerge worldwide with unpredictable pathogenicity, prompting continued awareness amongst clinicians on managing COVID-19 illness and its aftermath. Limitations of this case report are the lack of data available and reproducibility of the treatment effect. Pathologic findings in MIS-A may represent a heterogeneity of disease states and may not all respond to steroids and IVIg. Additionally, it is not clear which patient specific factors may predict a more favorable response to therapy.

MIS-A should be considered in patients presenting with fevers, elevated inflammatory markers, prior SARS-CoV-2 infection, severe dysfunction of one or more extra-pulmonary systems, and absence of severe respiratory illness. In this case, EMB facilitated identification of MIS-A and helped rule out acute fulminant myocarditis. Given rapid recovery of biventricular function with IVIg and steroids, the mechanism of cardiac injury is probably attributable to inflammation.

## Supplementary Information

**Additional file 1**. Supplemental Appendix.

**Additional file 2**. Supplemental Video 1a.

**Additional file 3**. Supplemental Video 1b.

**Additional file 4**. Supplemental Video 2a.

**Additional file 5**. Supplemental Video 2b.

## Data Availability

All data are available in the manuscript.

## Abbreviations

MIS-A: Multisystem inflammatory disorder in adults
COVID-19: Coronavirus disease 2019
EMB: Endomyocardial biopsy
SARS-CoV-2: Severe acute respiratory syndrome coronavirus 2
PCR: Polymerase chain reaction
IgG: Immunoglobulin G
NT-proBNP: N-terminal pro-B-type natriuretic peptide
ESR: Erythrocyte sedimentation rate
RA: Right atrium
RV: Right ventricle
PA: Pulmonary artery
PCWP: Pulmonary capillary wedge pressure
PAPi: Pulmonary artery pulsatility index
MAP: Mean arterial pressure
IVIg: Intravenous immunoglobulin
LVEF: Left ventricular ejection fraction
MIS-C: Multisystem inflammatory syndrome in children
