# Supplementary material for: Cardiogenic shock complicating multisystem inflammatory syndrome following COVID-19 infection: a case report
Source: BMC Cardiovasc Disord. 2021 Oct 29;21:522. doi: 10.1186/s12872-021-02304-y (PMC8555861; doi:10.1186/s12872-021-02304-y)
Supplement: Supplementary file 1 — Additional file 1. Supplemental Appendix. [file 12872_2021_2304_MOESM1_ESM.docx]

**Supplemental Appendix**

**Supplemental Video 1.** Echocardiogram with a) parasternal long axis view and b) four chamber view showing severely reduced global function, hypokinetic right ventricle, and biatrial dilation.

**
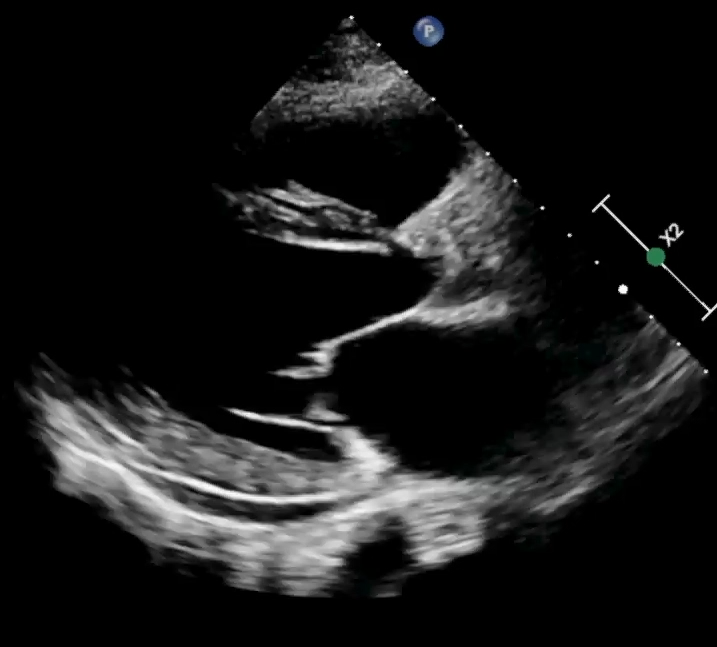
**a)

**
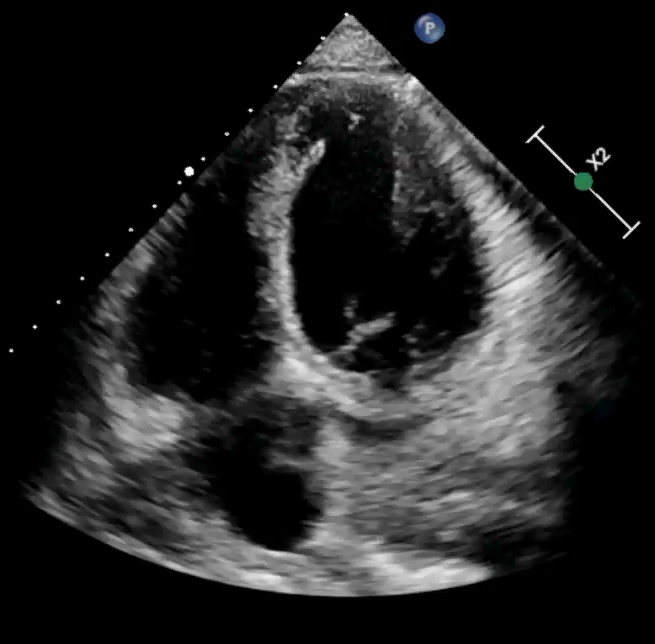
**

b)

**Supplemental Video 2.** Echocardiogram following treatment with IVIg and steroids a) parasternal long axis view and b) four chamber view (in chroma), with recovered EF, normal RV size and function, and small pericardial effusion. Pulmonary artery catheter present in the right heart.

**
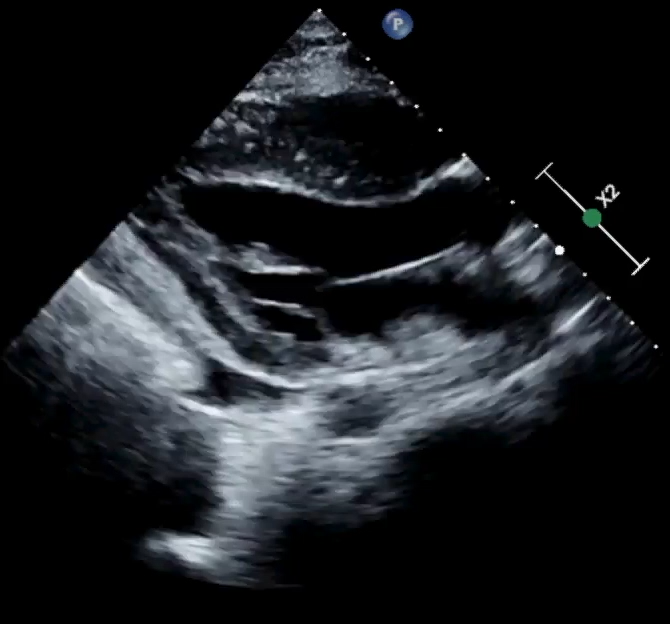
**

a)

**
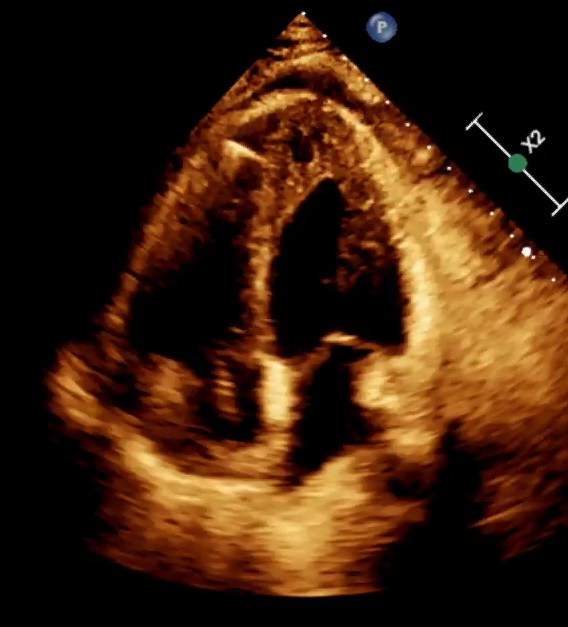
**b)
